# The Association between Individual SNPs or Haplotypes of Matrix Metalloproteinase 1 and Gastric Cancer Susceptibility, Progression and Prognosis

**DOI:** 10.1371/journal.pone.0038002

**Published:** 2012-05-24

**Authors:** Yong-Xi Song, Xin Zhou, Zhen-Ning Wang, Peng Gao, Ai-Lin Li, Ji-Wang Liang, Jin-Liang Zhu, Ying-Ying Xu, Hui-Mian Xu

**Affiliations:** 1 Department of Surgical Oncology and General Surgery, First Hospital of China Medical University, Shenyang, People's Republic of China; 2 Department of Gynecology and Obstetrics, ShengJing Hospital of China Medical University, Shenyang, People's Republic of China; Centers for Disease Control and Prevention, United States of America

## Abstract

**Background:**

The single nucleotide polymorphisms (SNPs) in matrix metalloproteinase 1(MMP-1)play important roles in some cancers. This study examined the associations between individual SNPs or haplotypes in MMP-1 and susceptibility, clinicopathological parameters and prognosis of gastric cancer in a large sample of the Han population in northern China.

**Methods:**

In this case–controlled study, there were 404 patients with gastric cancer and 404 healthy controls. Seven SNPs were genotyped using the MALDI-TOF MS system. Then, SPSS software, Haploview 4.2 software, Haplo.states software and THEsias software were used to estimate the association between individual SNPs or haplotypes of MMP-1 and gastric cancer susceptibility, progression and prognosis.

**Results:**

Among seven SNPs, there were no individual SNPs correlated to gastric cancer risk. Moreover, only the rs470206 genotype had a correlation with histologic grades, and the patients with GA/AA had well cell differentiation compared to the patients with genotype GG (OR=0.573; 95%CI: 0.353–0.929; P=0.023). Then, we constructed a four-marker haplotype block that contained 4 common haplotypes: TCCG, GCCG, TTCG and TTTA. However, all four common haplotypes had no correlation with gastric cancer risk and we did not find any relationship between these haplotypes and clinicopathological parameters in gastric cancer. Furthermore, neither individual SNPs nor haplotypes had an association with the survival of patients with gastric cancer.

**Conclusions:**

This study evaluated polymorphisms of the MMP-1 gene in gastric cancer with a MALDI-TOF MS method in a large northern Chinese case-controlled cohort. Our results indicated that these seven SNPs of MMP-1 might not be useful as significant markers to predict gastric cancer susceptibility, progression or prognosis, at least in the Han population in northern China.

## Introduction

Gastric cancer is one of the most common leading causes of cancer-related mortality worldwide [1]. Despite some advances in the diagnosis and treatment of gastric cancer in the last decades, the prognosis for patients with advanced gastric cancer remains poor [2]. Like other cancers, the development of gastric cancer is a multistep process with the accumulation of genetic and epigenetic changes. The discovery and application of biomarkers that can be incorporated with traditional cancer diagnosis, staging and prognosis could largely help to improve early diagnosis and patient care [3]. With the completion of the human genome project, millions of single nucleotide polymorphisms (SNPs) have been identified as attractive biomarkers in cancer risk assessment, screening, staging, or grading [4].

Matrix metalloproteinases (MMPs) are an important family of metal-dependent enzymes that are responsible for the degradation of extracellular matrix components [5]. Molecular epidemiologic studies have shown associations between genetic polymorphisms of MMPs and cancer susceptibility, progression and prognosis [6]–[10]. Recently, some SNPs of MMP-1 have been demonstrated to be significantly associated with increased risk for the development of lung cancer [6], [7], [11]. In breast cancer, Karolina Przybylowska et al. found that the 2G allele of the 1G/2G MMP-1 gene polymorphism may be responsible for lymph node (LN) metastasis [8]. On the other hand, both studies of Hinoda Y et al. and Ghilardi G et al. found that SNPs of MMP-1 were linked to an increased risk of colorectal cancer [12], [13]. Furthermore, a SNP in the MMP-1 promoter was demonstrated to be correlated with histological differentiation of gastric cancer [14]. However, other studies showed a negative association between MMP-1 polymorphisms and cancer susceptibility [15], [16], [17]. Furthermore, most of these studies were limited to small samples, few SNPs or constructed haplotypes from two or three polymorphic sites. Thus, a large sample and more polymorphic sites are critical to understanding the role of MMP-1 SNPs in gastric cancer development.

In the present study, in a large sample of the Han population in northern China, we used formalin-fixed, paraffin-embedded tissues (FFPETs)-derived DNA samples from patients and blood-derived DNA from controls in a matrix-assisted laser desorption/ionization time-of-flight mass spectrometry (MALDI-TOF MS) method to study the potential associations between seven SNPs (rs2071231, rs7125062, rs491152, rs470558, rs2075847, rs470206 and rs1144396) or haplotypes in MMP-1 and tumor susceptibility, clinicopathological parameters, and survival of gastric cancer.

## Materials and Methods

### Subject selection

This study consisted of 404 primary gastric cancer patients and 404 controls and all subjects were from the Han population in northern China. The subject characteristics have been described previously [18]. Briefly, eligible patients had received radical surgery at the First Hospital of China Medical University between January 1998 and December 2004 and were diagnosed with gastric cancer based on histopathological evaluation. The tumor histological grade was assessed according to World Health Organization criteria and tumors were staged using the 7th edition of the TNM staging of the International Union Against Cancer (UICC)/American Joint Committee on Cancer (AJCC) system (2010) based on postoperative pathological examination of the specimens. Complete pathological data were obtained including age, gender, date of surgery, location of the primary tumor, histologic grade, venous invasion, lymphovascular invasion, depth of invasion, number of LNs retrieved, number of metastatic LNs, and number of tumor deposits retrieved. Those (i) with synchronous or metachronous malignant tumors, (ii) with distant metastasis found preoperatively, (iii) who underwent preoperative radiotherapy or chemotherapy, or (iv) with incomplete pathological data entries were excluded from this study. Follow-up was completed for the entire study population until January 2010. Two patients died in the postoperative period (within 30 days) and 21 patients were lost to follow-up; therefore, 381 patients were included in survival analysis. Median and mean follow-up periods were 90.0 months and 93.3±20.24 months (range: 61–136 months), respectively. The following data were obtained for all patients: date of death (if applicable), cause of death (if applicable), and date of follow-up. The primary endpoint was cancer-specific survival duration from the date of gastric cancer diagnosis to the date of death. The 5-year survival rate of the 404 patients was 54.2%.

404 blood samples of the control group were obtained from cancer-free individuals that were randomly selected based on physical examinations during December 2009 to August 2011, and this group was believed to be a good representation of the population in this region. The selection criteria included no individual history of cancer, frequency matching to cases on sex and age and individuals were unrelated ethnic Han Chinese. The samples (Ethylene Diamine Tetraacetic Acid [EDTA] anticoagulate) were stored at −20°C within 30–40 minutes, and then moved to a freezer at −80°C within 2 or 3 days after collection.

### Ethics statement

The study was approved by the Research Ethics Committee of China Medical University, China. Written informed consent was obtained from all people before participating in the study.

### DNA extraction

Genomic DNA was extracted from FFPET samples in the case group. Sections with a thickness of 8 µm and a surface area of up to 250 mm^2^ were prepared with a microtome and DNA was isolated from 6 sections to 12 sections, depending on the tissue size and cell counts. The microtome was cleaned and the blades were changed to avoid intersample contamination. DNA extraction from FFPETs was performed with a QIAamp® DNA FFPE Tissue Kit (Qiagen, Hilden, Germany) [19], following the procedures described by the manufacturer and our previous work [18]. About 2–10 µg of DNA was recovered in 50 µl final solution and was stored at −80°C.

Genomic DNA was extracted from blood samples from the control group with the Universal Genomic DNA Extraction Kit Ver.3.0 (TAKARA) according to the manufacturer's instructions and our previous work [18]. About 2–6 µg of DNA was recovered in TE and was stored at −80°C.

### Selection of candidate SNPs

**Figure 1:**
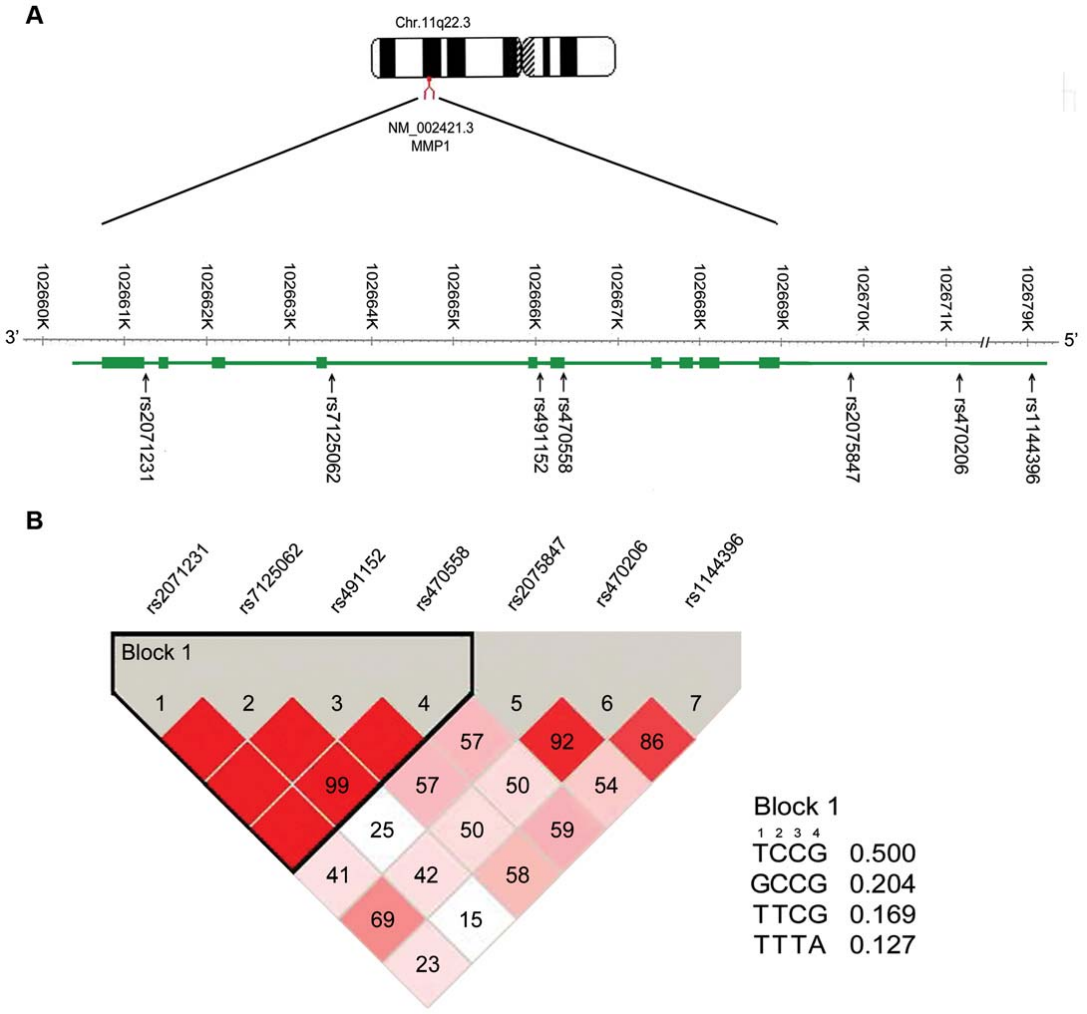
SNPs in the region of MMP-1 gene located in chromosome 11q22. **A**, MMP-1 gene structure. Filled boxes represent the exons (5′→3′). Arrows show the locations of SNPs. **B**: Mapping of the block structure of the seven SNPs generated by Haploview. The value within each square in the triangle plot represents the pairwise correlation between SNPs (measured as D′) defined by the upper left and the upper right sides of the Squares. The Squares without a number correspond to D′=1. Shading represents the magnitude and significance of pairwise LD, with a red-to-white gradient reflecting higher to lower LD values. The frequency of each common haplotype within a block is to the right of the haplotype.

The study included seven SNPs in MMP-1, which were taken from the NCBI SNPs database and the HapMap database. We selected SNPs across the gene loci to ensure a high density of markers and to provide adequate characterization of haplotype diversity [6]. All selected SNPs were required to have a minor allele frequency ≥5%. We therefore selected seven SNPs: rs2071231 (intron), rs7125062 (intron), rs491152 (intron), rs470558 (exon), rs2075847 (5′UTR), rs470206 (5′ UTR) and rs1144396 (Fig. 1A).

### SNPs analysis and validation

SNPs were genotyped using the MALDI-TOF MS system (MassARRAY; Sequenom, San Diego, CA, USA) with primers and probes (Table S1) as previously described [19], [20]. To ensure the typing quality, 1% positive samples (YanHuang cell strain) were incorporated into every genotyping plate to validate the reliability of the primers and 1% negative samples (water with no DNA) were used to monitor contamination. 5% random samples were tested in duplicate by different persons and the reproducibility was 100%. The laboratory personnel were blinded to the sample arrangement during the process. MALDI-TOF MS analysis were according to Justenhoven et al. [21] and the main process included PCR amplification (GeneAmp® PCR System 9700 Dual 384-Well Sample Block Module, Sequenom), shrimp alkaline phosphatase treatment (Sequenom), base extension reactions, salt removal with resin, SpectroCHIP dispensing (384-well SpectroCHIP microarray, Sequenom). Allelic discrimination was obtained by analysis with a MassARRAY Analyzer Compact mass spectrometer (MT9). Finally, data analysis were performed using MassArray Typer Analyzer software 4.0 (Sequenom, San Diego, CA) [22].

### Linkage disequilibrium (LD) block determination and haplotype construction

Haploview 4.2 software was used to evaluate LD and construct haplotypes as described previously [6]. LD between the seven SNPs used in haplotype analysis was measured by a pairwise D′ statistic. The structure of the LD block was examined using the method of Gabriel et al. [23], using the 80% confidence bounds of D′ to define sites of historical recombination between SNPs. Haplotypes were constructed from genotype data in the full-size case-control panel within blocks using an accelerated expectation-maximization algorithm method with Haploview 4.2 software [6], [24]. Furthermore, we made SNP genotype combinations to find their association with gastric cancer risk [25].

### Statistical analysis

A two-sided chi-square (χ2) test was used to estimate population distribution characteristics, compare differences in allelic and genotypic frequencies between cases and controls and estimate associations between individual SNPs and clinicopathological parameters. To assess significance, a permutation procedure (1,000 tests) was used to correct the P value of single-locus association results [6]. A permutation test is a type of statistical significance test in which the distribution of the test statistic under the null hypothesis is obtained by calculating all possible values of the test statistic under rearrangements of the labels on the observed data points. Moreover, the Bonferroni correction was used for multiple testing correction [26]. Logistic regression was used to analyze the association between genotype frequencies and gastric cancer risk, adjusted for sex and age. Survival analyses were done with the log-rank test and Cox proportional hazards model. The Haploview 4.2 software package was used to: estimate pair-wise LD, detect departure from the Hardy–Weinberg equilibrium, construct haplotypes, calculate haplotype frequencies and estimate associations between haplotypes and gastric cancer risk. We also used Haplo.states software to assess associations between haplotypes and clinicopathologic features [6], [27]. The THEsias software based on Cox proportional hazards survival regression in haplotype-based association analysis using the Stochastic-EM algorithm was used to produce survival analysis of haplotypes [28]. Because of multiple hypothesis testing, the P value for significance was adjusted conservatively by Bonferroni correction to <0.007 (0.05/7)

## Results

### Subject characteristics

As shown in Table 1 and previous work, the average age was 56.67±11.92 y and the percentage of males was 70.54% in the case group. The average age of the control group was 56.91±11.48 y and the percentage of males was 70.54%. There was no statistically significant difference in the distribution of sex and age between patients and controls (all P=1.00). Moreover, of the 404 patients, 85 (21.04%) had stage I gastric cancer, 107 (26.49%) had stage II gastric cancer, and 212 (52.48%) had stage III gastric cancer (Table 1). The other clinicopathological parameters of gastric cancer patients are shown in Table 1.

**Table 1:** Distributions of selected characteristics in gastric cancer cases and controls (n=404 for both case and control groups).

Variable	Patients (n=404) No. (%)	Controls (n=404) No.(%)	P^a^
Sex			1.0
Male	285(70.54)	285(70.54)	
Female	119(29.46)	119(29.46)	
Age at diagnosis			1.0
≤40	30 (7.43)	30 (7.43)	
41–50	94(23.27)	94(23.27)	
51–60	111(27.48)	111(27.48)	
61–70	122(30.20)	122(30.20)	
>70	47(11.63)	47(11.63)	
Tumor stage			
Ia	45(11.1)		
Ib	40(9.9)		
IIa	58(14.4)		
IIb	49(12.1)		
IIIa	57(14.1)		
IIIb	115(28.5)		
IIIc	40(9.9)		
IV	0(0)		
pT category			
T1	59(14.6)		
T2	70(17.3)		
T3	193(47.8)		
T4	82(20.3)		
Lymph node metastasis			
Negative	127(31.4)		
Positive	277(68.6)		
Borrmann type			
Borr1	64(15.8)		
Borr2	45(11.1)		
Borr3	266(65.8)		
Borr4	29(7.2)		
Histologic grade			
Well	94(23.3)		
Poor	310(76.7)		
Venous invasion			
Negative	400(99.0)		
Positive	4(1.0)		
Lymphovascular invasion			
Negative	303(75.0)		
Positive	101(25.0)		

a Two-sided χ^2^ test.

### Genotyping success rates, LD and haplotype structure

All the SNPs were polymorphic with minor allele frequencies >10% and genotype distributions were all in agreement with Hardy-Weinberg equilibrium (data not shown). The success rates were high in the case group (98.27–100%) and control group (99.50–100%; Table S2). Then, we used Haploview 4.2 software to evaluate LD and construct haplotypes. LD was observed across rs2071231, rs7125062, rs491152 and rs470558 (all D ≥0.99), and these four SNPs constructed block 1. Block 1 covered 5.0 kb and contained 4 common haplotypes: TCCG, GCCG, TTCG and TTTA (frequency range: 0.127–0.500), which represented approximately 99.9% of the subjects (Fig.1B).

### Associations between individual SNPs and gastric cancer risk, clinicopathological parameters and survival

As shown in Table 2, there was no statistical difference in the allele distribution between patients and controls (P>0.007 and P>0.007 after a permutation test for allelic frequencies and Bonferroni correction). Moreover, there was no association between genotype distributions of the seven SNPs in MMP-1 and the risk of gastric cancer (P>0.007 and P>0.007 after being adjusted for sex and age for genotypic frequencies, Table 3).

**Table 2:** Allele frequencies of seven SNPs in MMP-1 among patients and controls.

SNP^a^	Chromosome Position^b^	Allele	Risk allele	No.allele(%)		P^c^	P^d^
				Patients	Controls		
rs2071231	102661276	G/T	G	168(20.9)	159(19.7)	0.560	0.988
rs7125062	102663503	C/T	C	570(70.5)	568(70.3)	0.913	1.000
rs491152	102666043	C/T	T	103(12.8)	102(12.6)	0.925	1.000
rs470558	102666316	A/G	A	105(13.2)	102(12.7)	0.764	0.999
rs2075847	102669824	C/T	T	625(78.7)	612(75.7)	0.156	0.591
rs470206	102671178	A/G	A	132(16.3)	117(14.5)	0.301	0.862
rs1144396	102679052	A/C	C	536(66.3)	528(65.3)	0.675	0.996

a According to National Center for Biotechnology Information SNP database rs number.

b Chromosome Positions are from National Center for Biotechnology Information Build 37.3.

c Two-sided χ^2^ test.

d After 1,000 permutation tests.

**Table 3:** Associations between genotype distributions of seven SNPs in MMP-1 and the risk of gastric cancer.

Genotype	Controls	Patients	OR(95% CI)^a^	OR(95% CI) b	P a	P b
	No. (%)	No. (%)				
rs2071231	**403**	**402**			**0.482**	**0.481**
TT	266(66.0)	253(62.9)	1	1		
GT	115(28.5)	130(32.3)	1.189 (0.877–1.611)	1.189 (0.877–1.612)	0.266	0.265
GG	22(5.5)	19(4.7)	0.908 (0.480–1.718)	0.909 (0.480–1.720)	0.767	0.769
GT+GG	137(34.0)	149(37.1)	1.143 (0.857–1.526)	1.144 (0.857–1.527)	0.363	0.361
rs7125062	**404**	**404**			**0.839**	**0.824**
CC	200(49.5)	198(49.0)	1	1		
CT	168(41.6)	174(43.1)	1.046 (0.784–1.397)	1.046 (0.783–1.397)	0.760	0.763
TT	36(8.9)	32(7.9)	0.898 (0.536–1.503)	0.889 (0.530–1.490)	0.682	0.655
CT+TT	204(50.5)	206(51.0)	1.020 (0.774–1.344)	1.018 (0.772–1.342)	0.888	0.901
rs491152	**404**	**403**			**0.936**	**0.922**
CC	310(76.7)	307(76.2)	1	1		
CT	86(21.3)	89(22.1)	1.045 (0.747–1.462)	1.045 (0.747–1.462)	0.797	0.799
TT	8(2.0)	7(1.7)	0.884 (0.317–2.466)	0.857 (0.305–2.402)	0.813	0.769
CT+TT	94(23.3)	96(23.8)	1.031 (0.745–1.428)	1.028 (0.743–1.424)	0.853	0.866
rs470558	**402**	**398**			**0.864**	**0.853**
GG	308(76.6)	300(75.4)	1	1		
GA	86(21.4)	91(22.9)	1.086 (0.777–1.519)	1.086 (0.777–1.518)	0.628	0.629
AA	8(2.0)	7(1.8)	0.898 (0.322–2.508)	0.876 (0.312–2.456)	0.838	0.801
GA+AA	94(23.4)	98(24.6)	1.070 (0.774–1.481)	1.068(0.772–1.478)	0.681	0.690
rs2075847	**404**	**397**			**0.345**	**0.352**
TT	231(57.2)	244(61.5)	1	1		
TC	150(37.1)	137(34.5)	0.865 (0.645–1.159)	0.867 (0.647–1.163)	0.331	0.342
CC	23(5.7)	16(4.0)	0.659 (0.339–1.278)	0.659 (0.340–1.279)	0.217	0.218
TC+CC	173(42.8)	153(38.5)	0.837 (0.631–1.110)	0.840 (0.633–1.114)	0.218	0.225
rs470206	**404**	**404**			**0.314**	**0.319**
GG	292(72.3)	283(70.0)	1	1		
GA	107(26.5)	110(27.2)	1.061 (0.776–1.450)	1.060 (0.775–1.448)	0.711	0.717
AA	5(1.2)	11(2.7)	2.270 (0.779–6.616)	2.257 (0.774–6.580)	0.133	0.136
GA+AA	112(27.7)	121(30.0)	1.115 (0.822–1.512)	1.113 (0.821–1.510)	0.485	0.491
rs1144396	**404**	**404**			**0.883**	**0.888**
CC	169(41.8)	176(43.6)	1	1		
CA	190(47.0)	184(45.5)	0.930 (0.694–1.246)	0.930 (0.694–1.247)	0.626	0.629
AA	45(11.1)	44(10.9)	0.939 (0.589–1.496)	0.948 (0.594–1.512)	0.791	0.822
CA+AA	235(58.2)	228(56.4)	0.932 (0.705–1.231)	0.934 (0.706–1.234)	0.619	0.630

a Data were calculated by unconditional logistic regression.

b Data were calculated by unconditional logistic regression, and adjusted for sex, age.

Abbreviation: OR, odds ratio; CI, confidence interval.

The genotypes of individual SNPs were evaluated for associations with the clinicopathological parameters. Table 4 showed that rs470206 genotypes had a correlation with histologic grades, and the patients with GA/AA had well cell differentiation compared to the patients with genotype GG (OR=0.573; 95%CI: 0.353–0.929; P=0.023). However, it was not significant after Bonferroni correction. The other genotypes of SNPs had no significant correlations with clinicopathological parameters in gastric cancer.

**Table 4:** Associations between genotype distributions of the seven SNPs in MMP-1 and clinicopathological parameters.

	rs2071231		rs7125062		rs491152		rs470558		rs2075847		rs470206		rs1144396	
	TT	GT/GG	CC	CT/TT	CC	CT/TT	GG	GA/AA	TT	TC/CC	GG	GA/AA	CC	CA+AA
Borrmann type														
Borr1+2	71	38	46	63	81	28	79	28	61	46	77	32	39	70
Borr3+4	182	111	152	143	226	68	221	70	183	107	206	89	137	158
P^a^	0.577		0.096		0.592		0.664		0.268		0.874		0.055	
Histologic grade														
Well	60	34	44	50	66	27	65	28	54	39	57	37	39	55
Poor	193	115	154	156	241	69	235	70	190	114	226	84	137	173
P^a^	0.837		0.626		0.179		0.161		0.442		**0.023** b		0.643	
pT category														
T1	39	20	23	36	42	17	40	17	33	26	41	18	24	35
T2	37	33	38	32	56	14	56	14	40	25	47	23	30	40
T3	128	63	98	95	146	46	141	48	121	71	134	59	83	110
T4	49	33	39	43	63	19	63	19	50	31	61	21	39	43
P^a^	0.170		0.326		0.707		0.617		0.811		0.786		0.856	
Lymph node metastasis														
Negative	81	46	62	65	93	34	91	34	76	48	86	41	52	75
Positive	172	103	136	141	214	62	209	64	168	105	197	80	124	153
P^a^	0.812		0.959		0.346		0.419		0.962		0.488		0.472	
Venous invasion														
Negative	251	147	195	205	304	95	297	97	241	152	282	118	174	226
Positive	2	2	3	1	3	1	3	1	3	1	1	1	2	2
P^a^	0.629		0.363		1.000		1.000		1.000		0.082		1.000	
Lymphovascular invasion														
Negative	189	112	154	149	230	72	224	74	179	118	206	97	129	174
Positive	64	37	44	57	77	24	76	24	65	35	77	24	47	54
P^a^	0.917		0.206		0.987		0.867		0.401		0.117		0.487	
TNM stage														
I	54	31	45	40	61	24	59	24	46	37	58	27	31	54
II	65	41	52	55	82	25	82	25	68	37	76	31	53	54
III	134	77	109	103	164	47	159	49	130	79	149	63	92	120
P^a^	0.923		0.823		0.548		0.594		0.405		0.910		0.193	

a Two-sided χ^2^ test.

b OR=0.573; 95% CI: 0.353–0.929 comparison to the genotype GG.

In univariate analysis, Borrmann type, pT category, lymph node metastasis, lymphovascular invasion and TNM stage were demonstrated to be significant prognostic factors (Table 5). However, the statistical results revealed that all genotypes of the seven SNPs had no associations with survival of patients with gastric cancer (all P>0.007, Table 5).

**Table 5:** Univariate analysis of the prognostic factors for patients with gastric cancer.

	N^a^	5-YSR^b^(%)	P^c^
rs2071231			0.583
TT	237	53.9	
GT+GG	142	54.8	
rs7125062			0.240
CC	189	50.7	
CT+TT	192	57.6	
rs491152			0.150
CC	292	51.9	
CT+TT	88	61.0	
rs470558			0.150
GG	285	52.0	
GA+AA	90	60.7	
rs2075847			0.398
TT	229	52.4	
TC+CC	145	55.7	
rs470206			0.070
GG	266	51.0	
GA+AA	115	61.4	
rs1144396			0.821
CC	165	53.7	
CA+AA	216	54.5	
Age			0.175
≤60	229	57.1	
>60	152	49.6	
Sex			0.416
Male	272	55.2	
Female	109	51.6	
Borrmann type			<0.001
Borr1+2	103	81.6	
Borr3+4	278	43.8	
Histologic grade			0.441
Well	88	55.1	
Poor	293	53.9	
pT category			<0.001
T1	58	94.8	
T2	68	73.5	
T3	181	46.6	
T4	74	21.8	
Lymph node metastasis			<0.001
Negative	122	86.1	
Positive	259	38.7	
Venous invasion			0.280
Negative	377	54.5	
Positive	4	25.0	
Lymphovascular invasion			<0.001
Negative	287	60.1	
Positive	94	36.0	
TNM stage			<0.001
I	83	91.6	
II	101	74.2	
III	197	26.6	

a Number of patients

b 5-year accumulative survival rate

c P values were made by log-rank test

### Associations between haplotypes or SNP genotype combinations and gastric cancer risk, clinicopathological parameters and survival

Using Haploview 4.2 software, we constructed a four-marker haplotype block, which contained four common haplotypes: TCCG, GCCG, TTCG and TTTA. All four common haplotypes had no correlation with gastric cancer risk (P>0.007 and P>0.007 after a permutation test; Table S3). Moreover, we did not find any relationship between these four common haplotypes and clinicopathological parameters in gastric cancer (all P>0.007, Table S4). Furthermore, the result of univariate analysis showed no association between all the haplotypes and survival of patients with gastric cancer (all P>0.007, Table S5).

Then, we made SNP genotype combinations to find their association with gastric cancer risk. Among all combinations, genotype combinations of two SNPs (rs2071231 and rs470206) had four subgroups: TA, TG, GG and AA. Although the LD between the two SNPs did not exist and the four subgroups had no correlation with gastric cancer risk (P=0.523), the patients with TA had well cell differentiation compared to the patients with genotype TG (OR=0.561; 95%CI: 0.357–0.881; P=0.022). However, it was not significant after Bonferroni correction. Furthermore, all subgroups had no associations with survival of patients with gastric cancer (all P>0.007). The other genotype combinations had no significant results.

## Discussion

The degradation of extracellular matrix and basement membrane by MMPs is one of the most important regulatory elements in many physiological and pathological processes of tumor invasion and metastasis [5]. The majority of previous studies have focused on the relation between SNPs and MMP-2 and MMP-9. Furthermore, some SNPs of MMP-1 have been demonstrated to be significantly associated with increased risk for the development of lung cancer, breast cancer and colorectal cancer [6]–[8], [11]–[13]. However, other studies showed a negative association between MMP-1 polymorphisms and cancer susceptibility [15]–[17]. Moreover, in gastric cancer, most of the studies on MMP-1 have only focused on the importance of the SNP (−1607 1G/2G) in the promotor in relatively small samples. Jin X et al. reported that there was no association of the MMP-1 promoter polymorphism (−1607 1G/2G) with susceptibility to gastric cardiac adenocarcinoma in northern China (183 patients) [15]. However, a study on a Japanese population (215 patients) showed that, although the presence of the 2G allele (−1607) in the MMP-1 promoter did not enhance the risk of gastric cancer, it may be involved in differentiation of gastric cancer [14]. Hence, a large sample and more polymorphic sites are critical for understanding the role of MMP-1 SNPs in gastric cancer development.

Considering the above, we selected seven polymorphic sites across MMP-1 to ensure a high density of markers and to provide adequate characterization of haplotype diversity. Moreover, we selected 404 patients and 404 controls that had the same distributions for sex and age. On the other hand, until now, most of the SNPs were studied by restriction fragment length polymorphism (RFLP) assay [29], TaqMans [19], DHPLC [30], MALDI-TOF MS [6], [20] and pyrosequencing analysis [31]. Currently, the MALDI-TOF MS method, offering approximately 100% accuracy for SNP genotyping, is considered as a gold standard [19], [32]. Moreover, previous reports showed that there were no allelic frequency differences between FFPET-derived DNA and blood-derived DNA from the same individual through several methods, including MALDI-TOF MS [33]–[35]. Therefore, genotyping of FFPET-derived DNA by MALDI-TOF MS is reliable and reproducible. Our results also showed high success rates ranging between 98.27% and 100% (mean: 99.43%), which were in accordance with previous reported data [19], [32], [33].

Among seven SNPs, Sun et al. showed that risk allelic frequencies of rs7125062, rs2075847 and rs470206 were higher in patients with lung cancer than in controls [6]. But, in the present study, there were no individual SNPs correlated to gastric cancer risk. Moreover, rs470206 genotypes had a corelation with histologic grades, and the patients with GA/AA had well cell differentiation compared to the patients with genotype GG. This result was similar to the polymorphic sites (−1607 1G/2G), which may be involved in differentiation of gastric cancer [14]. Furthermore, some studies revealed that the MMP-1 promoter polymorphism (−1607 1G/2G) has an association with prognosis in tongue cancer [36], breast cancer [37] and colorectal cancer [38]. However, we did not find any individual SNPs correlated with prognosis in gastric cancer in our study.

SNPs are stably inherited, highly abundant and show diversity within and among populations, which are thought to be attractive biomarkers. However, the application of individual SNPs has been limited because they have low penetrance and their effects are relatively difficult to identify. Therefore, the importance of haplotype information has been increasing to link DNA sequence variation with disease [6], [18], [39]. In our study, we constructed a four-marker haplotype block that contained 4 common haplotypes: TCCG, GCCG, TTCG and TTTA, which were consistent with the study of Sun et al. [6]. Their study showed haplotype TTCG had a frequency that was significantly different between patients and controls. Moreover, haplotype TTCG had an increased risk for distant metastasis of lung cancer and, in contrast with haplotype TTCG, haplotype TTTA showed a protective effect against lung cancer progression [6]. However, in our study, all four common haplotypes had no correlation with gastric cancer risk and we did not find any relationship between these haplotypes and clinicopathological parameters in gastric cancer. Furthermore, the result of univariate analysis showed no association between all the haplotypes and survival of patients with gastric cancer. Until now, an increasing number of studies have focused on the association between SNPs and disease, but even with the same SNP, the results were usually different. More and more studies revealed that different results could be mainly attributable to various combinations of factors, such as disease heterogeneity, population, environment, allelic frequencies and/or LD differences, tissue source used, sample sizes, detection technique and so on.

Interestingly, although the LD between rs2071231 and rs470206 did not exist, we still made SNP genotype combinations according to the study of Ostrovsky O et al. [25]. We found that, compared to the patients with genotype TG, the patients with TA had well cell differentiation. However, the frequency of this type of SNP genotype combination was very low in the population.

In conclusion, this study evaluated polymorphisms of the MMP-1 gene in gastric cancer with a MALDI-TOF MS method and archived FFPETs in a large northern Chinese case-controlled cohort. Although our results were negative, this study first indicated that the SNPs (rs2071231, rs7125062, rs491152, rs470558, rs2075847, rs470206 and rs1144396) of MMP-1 might not be useful as significant markers to predict gastric cancer susceptibility, progression or prognosis, at least in the Han population in northern China. Moreover, these results could provide the significant information to other scientists doing cancer research to eliminate these seven SNPs as diagnostic markers for gastric cancer.

## Supporting Information

Table S1 Primer sequences used for genotyping the seven SNPs in MMP-1.
(DOC)

Table S2 Genotyping success rates of the seven SNPs in MMP-1.
(DOC)

Table S3 Associations between haplotype frequencies of four SNPs in MMP-1 and the risk of gastric cancer.
(DOC)

Table S4 Associations between haplotype frequencies of four SNPs in MMP-1 and clinicopathological parameters.
(DOC)

Table S5 Survival analysis of haplotypes of four SNPs in MMP-1.
(DOC)
