# Supplementary material for: The Association between Individual SNPs or Haplotypes of Matrix Metalloproteinase 1 and Gastric Cancer Susceptibility, Progression and Prognosis
Source: PLoS One. 2012 May 24;7(5):e38002. doi: 10.1371/journal.pone.0038002 (PMC3360011; doi:10.1371/journal.pone.0038002)
Supplement: Table S1 — Primer sequences used for genotyping the seven SNPs in MMP-1. (DOC) [file pone.0038002.s001.doc]

**Table S1.** Primer sequences used for genotyping the seven SNPs in MMP-1.

| **SNP** | **Forward primers** | **Reverse Primers** | **Extension primers** |
| --- | --- | --- | --- |
| rs2071231 | ACGTTGGATGCTTGTTCCATGAAAGAAATAG | ACGTTGGATGCCTAGAAACTACTTGTATAA | ATCCTAGAAACAAAACAAAAGAGACT |
| rs7125062 | ACGTTGGATGGATATCTGCCAGAGATTACC | ACGTTGGATGTTGTGCGCATGTAGAATCTG | CTGCCAGAGATTACCAGAAATT |
| rs491152 | ACGTTGGATGGTAGTTTTCTAATACTAGGC | ACGTTGGATGCTGGACAGGATTTTGGGAAC | TAATACTAGGCAAAACTAACTC |
| rs470558 | ACGTTGGATGATCAGTAGAATGGGAGAGTC | ACGTTGGATGTGGCCTTTTTAGAGTACAAC | AGTGGCCGAGTTCATGAGC |
| rs2075847 | ACGTTGGATGGTCCCATGATAATGATGGGC | ACGTTGGATGAGCCTTACCTGAGAAGACCC | ATCTCATACTCCGCCTG |
| rs470206 | ACGTTGGATGTGAGTCCAGAGCTCTCATGC | ACGTTGGATGCAACCAGTTTCCAATCCACG | TCCAGAGCTCTCATGCTAAATGCTCA |
| rs1144396 | ACGTTGGATGAGAAAGTGTAATGCGCTAAG | ACGTTGGATGGACGCTGCCGTCTTGTTACT | GTGAAACTATTGGGCTG |
