# Supplementary material for: The Association between Individual SNPs or Haplotypes of Matrix Metalloproteinase 1 and Gastric Cancer Susceptibility, Progression and Prognosis
Source: PLoS One. 2012 May 24;7(5):e38002. doi: 10.1371/journal.pone.0038002 (PMC3360011; doi:10.1371/journal.pone.0038002)
Supplement: Table S2 — Genotyping success rates of the seven SNPs in MMP-1. (DOC) [file pone.0038002.s002.doc]

**Table S2.** Genotyping success rates of the seven SNPs in MMP-1.

| SNP | Genotypes in FFPETs | | |  | Genotypes in Blood | | |
| --- | --- | --- | --- | --- | --- | --- | --- |
|  | Null (No.) | Total (No.) | Success rate (%) |  | Null (No.) | Total (No.) | Success rate (%) |
| rs 2071231 | 2 | 404 | 99.50 |  | 1 | 404 | 99.75 |
| rs 7125062 | 0 | 404 | 100.00 |  | 0 | 404 | 100.00 |
| rs 491152 | 1 | 404 | 99.75 |  | 0 | 404 | 100.00 |
| rs 470558 | 6 | 404 | 98.51 |  | 2 | 404 | 99.50 |
| rs 2075847 | 7 | 404 | 98.27 |  | 0 | 404 | 100.00 |
| rs 470206 | 0 | 404 | 100.00 |  | 0 | 404 | 100.00 |
| rs 1144396 | 0 | 404 | 100.00 |  | 0 | 404 | 100.00 |
| Mean | 16 | 2828 | 99.43 |  | 3 | 2828 | 99.89 |
