# Supplementary material for: The Association between Individual SNPs or Haplotypes of Matrix Metalloproteinase 1 and Gastric Cancer Susceptibility, Progression and Prognosis
Source: PLoS One. 2012 May 24;7(5):e38002. doi: 10.1371/journal.pone.0038002 (PMC3360011; doi:10.1371/journal.pone.0038002)
Supplement: Table S3 — Associations between haplotype frequencies of four SNPs in MMP-1 and the risk of gastric cancer. (DOC) [file pone.0038002.s003.doc]

**Table S3.** Associations between haplotype frequencies of four SNPs in MMP-1 and the risk of gastric cancer (n=404 for both case and control groups).

| Haplotype | All subjects | Patients | Controls | P^a^ | P^b^ |
| --- | --- | --- | --- | --- | --- |
| Block 1 |  |  |  |  |  |
| TCCG | 0.500 | 0.495 | 0.505 | 0.680 | 0.977 |
| GCCG | 0.204 | 0.210 | 0.197 | 0.532 | 0.913 |
| TTCG | 0.169 | 0.167 | 0.171 | 0.825 | 0.997 |
| TTTA | 0.127 | 0.128 | 0.126 | 0.910 | 1.000 |
| Overall |  |  |  | 0.646 | 0.653 |

^a^Two-sided χ^2^ test, each haplotype compared with all other haplotypes.

^b^After 1,000 permutation tests.
