# Supplementary material for: The Association between Individual SNPs or Haplotypes of Matrix Metalloproteinase 1 and Gastric Cancer Susceptibility, Progression and Prognosis
Source: PLoS One. 2012 May 24;7(5):e38002. doi: 10.1371/journal.pone.0038002 (PMC3360011; doi:10.1371/journal.pone.0038002)
Supplement: Table S4 — Associations between haplotype frequencies of four SNPs in MMP-1 and clinicopathological parameters. (DOC) [file pone.0038002.s004.doc]

**Table S4.** Associations between haplotype frequencies of four SNPs in MMP-1 and clinicopathological parameters (n=404).

| **Block1** | | | | | |
| --- | --- | --- | --- | --- | --- |
|  | **TCCG** | GCCG | TTCG | TTTA | overall |
| Borrmann type |  |  |  |  |  |
| Borr1+2 | 0.491 | 0.183 | 0.193 | 0.133 |  |
| Borr3+4 | 0.495 | 0.219 | 0.158 | 0.125 |  |
| P^a^ | 0.891 | 0.283 | 0.189 | 0.775 | 0.532 |
| Histologic grade |  |  |  |  |  |
| Well | 0.489 | 0.197 | 0.154 | 0.154 |  |
| Poor | 0.495 | 0.213 | 0.171 | 0.119 |  |
| P^a^ | 0.863 | 0.642 | 0.578 | 0.211 | 0.622 |
| pT category |  |  |  |  |  |
| T1 | 0.483 | 0.169 | 0.194 | 0.153 |  |
| T2 | 0.471 | 0.271 | 0.150 | 0.107 |  |
| T3 | 0.516 | 0.189 | 0.161 | 0.130 |  |
| T4 | 0.470 | 0.231 | 0.177 | 0.122 |  |
| P^a^ | 0.931 | 0.830 | 0.909 | 0.751 | 0.996 |
| Lymph node metastasis |  |  |  |  |  |
| N0 | 0.500 | 0.201 | 0.157 | 0.142 |  |
| N1+N2+N3 | 0.491 | 0.213 | 0.171 | 0.121 |  |
| P^a^ | 0.824 | 0.699 | 0.615 | 0.413 | 0.768 |
| Venous invasion |  |  |  |  |  |
| Negative | 0.493 | 0.209 | 0.169 | 0.128 |  |
| Positive | 0.625 | 0.250 | 0.000 | 0.125 |  |
| P^a^ | 0.456 | 0.775 | 0.203 | 0.983 | 0.740 |
| Lymphovascular invasion |  |  |  |  |  |
| Negative | 0.508 | 0.208 | 0.157 | 0.128 |  |
| Positive | 0.450 | 0.213 | 0.198 | 0.139 |  |
| P^a^ | 0.125 | 0.878 | 0.132 | 0.585 | 0.403 |
| TNM stage |  |  |  |  |  |
| I | 0.506 | 0.188 | 0.159 | 0.147 |  |
| II | 0.475 | 0.230 | 0.164 | 0.131 |  |
| III | 0.499 | 0.207 | 0.172 | 0.118 |  |
| P^a^ | 0.908 | 0.890 | 0.679 | 0.343 | 0.600 |

^a^Two-sided χ^2^ test, each haplotype compared with all other haplotypes.
