# Supplementary material for: The Association between Individual SNPs or Haplotypes of Matrix Metalloproteinase 1 and Gastric Cancer Susceptibility, Progression and Prognosis
Source: PLoS One. 2012 May 24;7(5):e38002. doi: 10.1371/journal.pone.0038002 (PMC3360011; doi:10.1371/journal.pone.0038002)
Supplement: Table S5 — Survival analysis of haplotypes of four SNPs in MMP-1. (DOC) [file pone.0038002.s005.doc]

**Table S5.** Survival analysis of haplotypes of four SNPs in MMP-1.

|  | **Frequencies** | **HR(95%CI)** | **P^a^** |
| --- | --- | --- | --- |
| Block1 |  |  |  |
| TCCG | 0.497 | 1 |  |
| GCCG | 0.211 | 0.929(0.699-1.235) | 0.613 |
| TTCG | 0.164 | 1.015(0.750-1.375) | 0.922 |
| TTTA | 0.124 | 0.756(0.536-1.067) | 0.111 |

^a^Based on Cox proportional hazards survival regression in haplotype-based association analysis using the Stochastic-EM algorithm.

Abbreviation: HR, hazard rate; CI, confidence interval.
